# The Sanatary Characteristics of Chicago, 1853

**Published:** 1854-02

**Authors:** N. S. Davis


*The Sanatary Characteristics of Chicago*, 1853. Being a Report read to the Cook Co. Medical Society, Feb. 7th, 1854. By N. S. Davis, M.D., &c. &c.

Few things would contribute more to the real advancement of practical medicine, than a careful and accurately kept record of diseases and their peculiarities in connection with the meteorological and climatic features of each season. That all diseases are susceptible of changes in their specific character, tendencies, and results, is fully proved by the recorded experience of the past. This is true of diseases, not only in reference to their prevalence in different locations and different climates, but also in reference to their prevalence in the same place at different seasons and periods of time. It has been often and justly remarked, that these variations in the character of diseases in different places, and in the same place at different times, have led to much of the seeming contradictory experience detailed by different writers, and the consequent confusion of opinions in relation to the indications for treatment and the value of remedial agents.—That variations in the special character of any given disease, are produced in isoluted or individual cases, by circumstances of a strictly local or artificial nature there can be no doubt. To this class of circumstances belong peculiarities of temperament, habits of life, imperfect ventilation, unwholesome food, &c. But beyond all these, we see in the same place, with no *visible* change either in the habits of the people or the physicial condition of the place itself, the prevalence of severe and fatal diseases during one season, and their entire absence another, constituting epidemics of irregular appearance, and also decided differences in the number and severity of ordinary sporadic attacks. Thus in this city since the summer of 1849, we have seen epidemic Cholera re-appear with each returning summer until that of 1853, when it was almost, if not entirely, absent. Yet during the latter season there existed *apparently* the same causes and physical circumstances as during each of the preceding years.—No material change had been made in the habits of our citizens, the cleanliness and drainage of our streets, or any of the sanatary regulations of our city.—Still, unless we are prepared to admit that sickness and pestilence are sent by an arbitrary and direct act of an over-ruling Providence, we must suppose that every effect has its cause; and consequently every change in the former is preceded by a corresponding change in the latter. That variations in the character of ordinary diseases, and the occurrence of irregular epidemics, are dependent on changes in the more occult or invisible elements of the atmosphere, such as its temperature, moisture, and electricity, has long been the opinion of medical men in every part of the world. But even up to the present time no adequate means have been devised for keeping a reliable note of the condition of all these elements in direct connection with the prevalence and character of diseases, through successive years in the same place, or in places remote from each other during the same year.—The partial attempts which have hitherto been made to effect this object have included scarcely more than a record of the *temperature*, leaving the equally important elements of moisture and electricity either wholly unnoted, or so imperfectly as to render the results of no value.—Indeed, until a very recent period, the instruments necessary for measuring the moisture of the atmosphere at short and regular intervals of time, have been too imperfect for practical use. Recently, however, it has been found that the actual *humidity* of the atmosphere can be more readily and accurately ascertained by the Wet-bulb Thermometer, than by the various Hygrometrical and Dew-point instruments previously in use.—I think we may reasonably expect that the adoption of this simple instrument, will soon give us as reliable a record of atmosphere humidity as we now have of temperature; and as the electrical condition is doubtless dependent more or less directly on those of temperature and humidity, we shall have a much more complete knowledge of all the elements that enter into the climat features of different seasonsic and localities. Previous to the comencement of 1853, the Smithsonian Institute had placed in the hands of instructed and competent agents (uniform instruments,) located in so many sections of the country as to afford a good index for the whole, and required of them quarterly or monthly returns. The first published results of these returns that have come under my observation, are contained in an article in the November No. of the New York *Journal of Medicine*, by Lorin Blodget, who has charge of the Meteorological Department of the Institute. And I cannot better convey an idea of the importance and practical bearing of these records, than by quoting the results in regard to the last preceding summer months in his own language, as follows, viz :—

"The summer of 1853 has been extraordinary in its climatic conditions, and the extremes of temperature and humidity were much more striking and clearly defined than usual. The relation which these conditions may have to the general sanatary character of a month or season, may therefore be analyzed under unusually favorable circumstances, if our observation are sufficient to define them with precision. The purpose of this paper is to present them in the proper grouping and contrasts, and this process will serve as a general test of their accuracy, and apply them to their most important purpose at the same time.

Some of the more disastrous periods of mortality of the summer have been attributed directly to extreme temperature, while the attendant condition of humidity has scarcely been recognized as influencing results. Violent epidemics, and even contagious diseases, may have been initiated by some maximum of these conditions, and the subsequent propagation and continuation may be due to other causes more difficult to decide upon. Without assuming entire dependence upon the conditions of climate we now measure for epidemics of the more violent character, it is still a point gained of unusual importance when numerical values may be given to humidity as well as temperature. With these the statistics of disease and mortality may ultimately undergo thorough comparison and examination, and to that research the greatest and final results will belong.

The climatic research conducted by the Smithsonian Institution now embraces every climatic district of the country. The full registers, including with the more common observation the difficult one of the temperature of evaporation, from which is deduced the relative humidity, number about thirty, which are immediately returned at the close of an observed month. There are others in the New York and Military System, accessible at a latter period ; but the first suffice to determine every important district in this respect. Correct temperature observations for the open air are reported in the same prompt manner from about two hundred stations, of which about fifty measure carefully the amount of precipitation

From these data the present exhibit is prepared. It will present, first, the several periods of excessive heat in the three summer months of the present year, and the comparison of various districts during those periods.

Second, the periods of excessive humidity, and of unusual amount of precipitation; and the relation of these in time and place to the division of excessive variations of temperature.

It also marks extremes of absence of humidity, and of cold, which are less important as santaary conditions standing alone, though the low humidity apparently occurs generally as very important modification of the injurious character of a period of excessive heat.

The first general high temperature of the season occured on the 3d to the 5th of June, extending from Montreal to Florida, but this did not occur at the West generally. At the South it was a day later than in New York, and its maximum at the North, from Montreal to New York, 83°, and from Chapel Hill, N. C., to Savannah, Ga., 92°. The period was so short that no general consequence would probably attach to it, however, and the amount of humidity was not unusual.

From the 14th to the 18th, the heat was excessive and general. At the extreme West, in Minnesota, Iowa and Wisconsin, it occurred on the 12th and 13th; the atmosphere affected passing eastward across the continent as a well-defined body, according to what seems a law of all dynamic or inconstant climatic phenomena. It did not extend beyond Camden, S. C., southward, and attained a higher point at the West than elsewhere, of 90° to 94° in Ohio, Kentucky, and westward in the same latitudes. Succeeding this, a general excess of temperature occurred from the 20th to the 23d, less than the preceding in the extreme North, and with a considerable fall there on the 22d, but quite unusual and long-continued at almost every other part of the country. The maxima varied from the 20th to 23d, and ranged from 90° to 97°. With the slight allowance for excess, which usually should be made in the readings at these extreme points, the maximum of 95° was probably general, from New York to Savannah, on the 23d, and the same degree the day previous in Ohio and Michigan In Tennessee and southwestward, the same point was reached on the 20th; in Mississippi and Texas still one day earlier.

Lastly, a most extraordinary extreme of heat occurred on the 27th and 30th; In this case, as before, beginning earliest at the West by a day and a half for the distance from St. Louis to Washington. This extreme was central in the latitude of Washington, and was limited at Savannah on the South, and Burlington, Vt., on the North. It attained 96° to 98° in Tennessee, Kentucky, and Southern Ohio, and 99.5° to 102° at Washington and in Eastern Virginia and N. Carolina. This is without any known parallel in the records of temperature here, and is several degrees above any recorded temperatures at New Orleans, Mobile, or Savannah.

The general temperature of July was also high, and slightly above the normal mean in most parts of the United States. The mean of June was much above the normal, one, attaining a maximum of excess in Wisconsin and Illinois of nine degrees. It was there 4.5° above the mean for July,—this excess being less at the East, where the two months were nearly equal, and June 5° above the normal mean.

The excessive heat of the last days of June was prolonged through the 1st and 2d of July at 94° in Virginia and at the South, and the range was generally high at Philadelphia and South until the 10th, when it was again at 92° to 84°. North of latitude 40° there were no great heats in this month, and the temperature was quite uniform throughout. The temperature was at or above 90, after the middle of the month, only in Central Georgia and Alabama, and westward in this latitude to Texas, for two or three days about the 20th, and again at the close of the month.

In August, a period of general excessive heat occurred, beginning, as usual, earlier at the West, and reaching 90° at Lac-qui-parle, on the St. Peter's River, Minnesota, (lat. 45°, long. 96°,) on the 7th and 8th. The maximum in Illinois and the adjacent States was 90° to 94° from the 8th to the 13th, in Ohio and Kentucky nearly the same ; and, passing eastward a little later through Pennsylvania, the district of greatest excess was central at New York from the 12th to the 14th.

The temperature was below 80° at Cedar Keys, Talahassee, and Pensacola, Fla., through these days, and at no place South reached 90°. Later in the month, from the 25th to the 31st, the heat was unusually great in the Southwest,—Texas, the Cherokee Territory, and Mississippi,—with an extraordinary reverse in Iowa and the adjoining States. Frosts occurred in all the States West and North of Ohio at some day from the 25th to 28th inclusive ; and in some parts of Iowa, Wisconsin, and Michigan, there were two or three in succession, and great injury was done to vegetation. At Lac-qui-parle, Minnesota, 8° farther north, and nearly a thousand feet higher, there was one very slight frost on the 27th.

These extraordinary conditions of temperature become more important when connected with the accompanying conditions of humidity. As general comparisons of this last condition have hardly been possible heretofore, the present summer cannot be compared with previous years, except in one or two extremes of its sanatary effect.

The observations of humidity are, however, believed to be of the very best character. The temparature of the *natural evaporation* has been taken under uniform directions, and with uniform instruments. The actual *evaporating effect* of the air at the time, as the measure of its true evaporating power in a climatic sense, and such as could affect animal life and the growth of vegetation, has been sought in the directions given, and in the practice of the observers.

From such observations the fraction of saturation, or the percentage of humidity existing in the air at the time, has been deduced by Regnault's formulas, first determined theoretically from the latent and specific heat of air and aqueous vapor, and subsequently modified in the numerical elements to conform to the results derived from actual experiment.

These results are arranged in a tabular form for greater convenience of comparison. The "relative humidity" is the percentage of moisture which the air at the time contains. 100 being full saturation. The absorbent power of the air in highly heated periods is directly as this decrease of the degree of saturation, and is the most precise measure possible of the sanatary condition which is peculiarly climatic, and not dependent on miasmatic or other secondary causes.

A brief reference to the prominent points in this comparison may assist the examination. It may be stated that the fractions of saturation are much more variable in cold than in warm climates at all temperatures. The mean summer saturation at New-Orleans, by the valuable observations of Dr. Barton,* is .857, and the mean saturation for the year .811. The degree here is quite constant through successive years for the different seasons, and has its maximum in summer. The maximum in the northern portion of the United States is also in summer, but the mean is something more then one-tenth of saturation less than that at New-Orleans.

The fractions given in the tabular statements used for the present general comparisons are from one observation only for the day, that at 2 P. M.; which is very near the hour for maximum temperature also. It would be equally important to give the mean of the day, but as the labor would be very much increased, the single hour of extreme range of the several conditions, giving the most striking contrasts, has been thought sufficient for the present purpose.

The heats of June are seen at once to have been remarkably dry. The fraction of saturation was at a mean of about 50 in the North-east, and but 40 to 45 in the interior and in Texas, during the hot days from the 14th to the 17th, though much higher at Pensacola and the extreme South, where the heat was not so great.

* *Report to the La. State Med. Soc., on the Meteorology, Vital Statistics, and Hygiene of Louisiana.* By Dr. E. H. Barton, 1851. The amounts here given are a summary from observations at sunrise, midday and 9 P. M., for eight years.

At the 20th to the 23d, the rate was about the same in the districts of excessive heat. On the 29th and 30th, the percentage was but 35 to 40 in the narrow district through Tennessee, Kentucky, and Virginia, which marked 100° as the maximum temperature. The coincidence of absence of humidity with this unequalled temperature was fortunate. The usual saturation with this degree of heat would have been extremely destructive to health and life.

The first two days of July were a continuation of the conditions of the last of June. The remainder of the month was not unusual in its hygrometric character generally, though at New-Orleans, where, unfortunately, we have no observations after May, the evidences of high saturation are given in the profuse and constant rains of the middle of the day, preceded by a hot and oppressive morning. This weather was reported as quite uniform for the first and most fearfully fatal month of the yellow fever. The approach to a tropical character in the midsummer saturation and daily rains, at New-Orleans, is more or less distincly marked in different years at all times, but the present summer seems to have attained a maximum in this respect, and to have given in this way terrible effect to the epidemic, when once induced, in whatever manner that may have been. It is hoped that careful observations of the humidity of the yellow-fever period of the present summer there, have been made. The Gulf Coast in Texas and Florida does not properly represent the humidity or other climatic peculiarities of New-Orleans, nor does any place in the interior from which we have observations.

The great heat of August was most remarkable in its hygrometric condition also, and universally attended with a high fraction of saturation. At Washington it was 55 to 50, and at New York near 70 per cent. at 2 P. M., and almost at saturation morning and evening. Though the temperature of the air was but 90° to 94°, or 6° less than that of the last of June in Washington and Richmond, where no fatal effects were felt, the mortality from the effect of great heat with great saturation was frightful. By reference to the tabular statement it may be seen that the absolute degree of heat could not have caused the fatality alone, or without the attendant humidity. Some term more specific or more comprehensive than *sun-str oke* seems therefore required to designate the fatal congestion, or whatever may be the immediate cause of death, in these cases.

Another and perhaps clearer point for comparison is the *temperature of evaporation*, or that of the wet-bulb thermometer alone. The dew-point has usually been obtained for such comparisons, but it is far less easy to obtain without error; and, as it is itself no absolute determination, but an intermediate point only, it is quite generally discontinued.

The temperature of evaporation at New York, at the time of greatest mortality in August, was from 80° to 84°, being higher than the maximum temperature of evaporation at New-Orleans at any time in 1852 by two degrees. At the latter place it reached 82° but once in that year. At Austin, Texas, for the last August, the same maximum was 78°; at Savannah, Ga., 79°; at Camden, S. C., Culloden, Ga., and Washington, 77°. Jacksonville, Fla., and Eutaw, in Central Alabama, were the only places at the South giving a temperature of evaporation of 84°, or as high as that at New York on the 13th and 14th of August.

With such conditions of temperature and saturation, in a nominally elastic and cool climate, it is not wonderful that the mortality from its immediate effect should have been very great. It is more remarkable that some incipient epidemic was not started in full vigor to rage after the principal inducing cause had passed away. With the exceptions of New-Orleans, and New-York at this limited period, the heats of the summer, though extreme, have been attended with a low humidity, and have been unusually favorable in this respect.

From this lengthy extract it will be seen, that the three summer months of 1853 were considerably above the mean temperature of the same months for a series of years. This was more particularly true of the months of June and August; both being throughout the North-west, from 3° to 5° above the usual standard. But fortunately in the same section of country, both months were attended by a low degree of *humidity*. The unusual dryness of the atmosphere was general over the whole Union during the high temperature in June, but not so in August.

During the excessive heat in the latter month, the *humidity* at New York, and along most of the Atlantic and Gulf coast, was very high, approximating closely the point of saturation, while in the North-west it still remained low. During the rest of the year I have no other date in reference to the climatic features, than such general facts as were noted in my own memorandum book. These, however, will be alluded to in direct connection with a brief account of the diseases of the past year, to which I will now call your attention. The first half of January, 1853, was warm and wet; the roads much of the time being sloppy; but the last half was cold and dry, the mercury in the barometer often standing at 30 inches.

Early in this month I noticed the frequent occurrence, especially among children, of an eruptive influenza or miliary fever, so closely resembling scarlatina in some respects as to have been very generally confounded with that disease. Its onset was generally characterized by a quick pulse, hot skin, restlessness, thirst, pains in the head, back and limbs, and soreness of the fauces and pharynx, with more or less swelling of the glands behind the angle of the jaw. So far the cases closely resembled scarlatina. But the eruption was more decidedly papular, and the redness of the skin less diffused than in the latter disease; and the eruption was also extremely irregular in regard to the time of its appearance. In some cases the appearance of the eruption was among the first symptoms of ill-health, while in a larger number it did not appear until the 4th, 5th, 6th, and even 7th day after the commencement of the fever. The disease generally ran its course in from one to two weeks, leaving considerable debility and sometimes a tedious convalescence. The mildest cases required no other treatment than a restricted diet rest, and the avoidance of all exposure. The more severe cases were promptly moderated in their violence by the exhibition of a powder composed of sub. murias hydrarg. from one to two grains, and pulv. doveri from two to five grains, given every three or four hours, until five or six doses had been taken, and then followed by a laxative of castor oil, or rhubarb and soda. If the urinary secretion was very scanty, half a tea-spoonful of spts. nit. dulc. was given between each of the powders; and if much swelling of the glands of the neck or soreness of the throat existed, cloths were applied wet in infusion of hops or aconite leaves. After the foregoing treatment, it was generally sufficient to give the patient from half a drachm to a drachm of the following mixiture from three to six times in the twenty-four hours—viz:

℞ Hive Syrup—ʒij.

Tinct. Belladona—ʒj.

Spts. Nit. Dulc.—℥jss M.

and convalescence would ensue in a few days. The disease did not bear active evacuating remedies without injury. The only cases I saw that resulted in great debility or protracted swelling of the cervical glands, or were followed by symptoms of acute dropsy, had been actively purged, or vomited, or both in the early stage of the disease. I saw only two or three cases during the month, that presented the full characteristics of scarlatina. The disease before alluded to, continued to prevail moderately during the month of February, and the fore part of March. The whole number of cases was such as to entitle it to the appellation of a mild epidemic. The months of March, April, and May were as usual changeable and wet. But they were not marked by the prevalence of any special diseases, though the occurrence of chronic and relapsing intermittents were more frequent than at the corresponding period of the three previous years. During the last half of March, my notes show the occurrence of several attacks of cholera morbus, two of which put on all the characteristic symptoms of sposmodic cholera, viz.: Rice-water discharges from the stomach and bowels, cramps in the lower extremities and abdomen, corrugated and purple skin, husky voice, sunken eyes and feeble pulse, etc. Both however recovered. Several of the milder cases were disposed to put on the form of dysintery after the first three or four days, accompanied by fever and tenesmus. These attacks become less frequent during the first half of April, and in their place I met with several cases of Remittent Fever, three of which assumed a highly pernicious or congestive form. The last half of April, and the whole of May were attended by no special prevalence of disease. Rains were frequent, and during the first half of May the surface soil was thoroughly saturated with fresh water, and the atmosphere was cool. The latter quality continued with but little rain until the first week in June. From that time to the first of September, the peculiarities of the weather have been accurately detailed in the quotation already given. Notwithstanding the unusual high temperature of June, no severe sickness was developed in this city or throughout the country generally. Sporadic cases of dysentery, typhoid fever, and occasional cases of intermittent and remittent fevers were met with during that month, and still more frequently during the months of July and August. Nothing unusual was noticed in reference to the fevers. They were generally mild and readily controlled by proper treatment. The dysenteries presented generally a quicker and sharper pulse, more heat of skin, more tenesmus, and discharges composed more of glairy mucus streaked with blood in the first stage, and more mixed with whitish flakes and purulent matter at a latter period, than during the three preceding summers. In other words, the symptoms of local inflammation were more acute or *stenic* in their character, and consequently would not tolerate us early a resort to astringents and tonics. During the last half of August and whole of September, more than double the number of intermittents and remittents were met with, than during the same months of 1850, '51 and '52, when epidemic cholera was prevalent in the city. From what we have learned, the same remark is applicable to the North-west generally.

The contrast, however, is not owing to an unusual prevalence of periodical fevers during the past summer and autumn, but to their unusual absence during the three preceding seasons when cholera was prevalent, and in many places epidemic. Several cases occurred at the foot of South-water street, directly on the Lake shore, a location generally entirely exempt. This was doubtless owing to the recent filling up of a pier projecting into the Lake, and preventing the usual washing of the waves on the beach. And it may well lead to a fear, that the present break-water of the railroad may materially deteriorate the healthiness of the whole Lake shore along the south division of the city. During the summer months, six or seven cases of cholera occurred under my own observation, but entirely disconnected from each other. Two of these were admitted into the Mercy Hospital. Of the whole number two died in a state of collapse, one preceded a few hours by hemorrhage from the bowels. Three others assumed the form of protracted dysentery after reaction was established, but they ultimately recovered. The disease at no period manifested a disposition to spread or assume an epidemic form. The month of October was dry, pleasant and healthy. The last half of November, and nearly all of December, were warm and wet, with the atmosphere almost constantly saturated with moisture. During this time diarrhœas, dysenteries, relapsing agues, and typhoid fevers became decidedly more prevalent; but they were generally mild in character and consequently added but little to the general ratio of mortality.

From the 20th of November to the end of the year, I met with one form of disease, so often that, I think it justly entitled to the name of epidemic. And from its rare appearance as a prevalent disease, it merits a more particular notice. Its most striking feature was a deep yellow, or jaundiced hue of the whole cutaneous surface, extending to the conjunctiva, and also to the mucous membrane lining the mouth. An account of the occasional prevalence of jaundice in an epidemic form may be found in the literary records of our profession; but such occurrences have been rare. The disease to which I have alluded as prevailing during the last six weeks of the past year, was generally characterized by the following symptoms, viz:—an impaired appetite; an unpleasant, often bitter taste in the mouth; a lassitude or feeling of indisposition with dulness or drowsiness; an unusual sense of distension in the epigastrium after taking food; skin drier than natural; bowels sometimes slightly relaxed, but more frequently moderately constipated; and the conjunctiva moderately yellow. These symptoms would appear very slight at first, and gradually increase, until at the end of ten days, or two weeks, when the skin and eyes would generally appear deeply yellow; the urine scanty and high colored; the tongue covered with a yellowish white fur; the pulse slightly quickened; the skin dry and warmer than natural; the appetite and digestion more decidedly deranged; the mental dulness greater; with a constant feeling of uneasiness or dull pain or sense of fulness in the epegastric and right hypochondriac regions; and obscure tenderness to pressure directly over the commencement of the duodenum.

In many cases the pressure over the epegestrium or beneath the cartilaginous margin of the ribs of the right side, would induce, not only tenderness, but also an immediate feeling of sickness or nausea. The fecal evacuations were generally consistent and clay colored, though in some instances they were liquid and sudsy, or of a dirty white color. In every instance that I examined they were deficient in the coloring matter of the bile, while the urine, the cutaneous exhalation, and the saliva were deeply tinged with the same ingredient. Occasionally, in addition to the mental dulness or drowsiness, slight wandering of thoughts or passive delirium was observed, especially at night. If not actively interfered with by medical treatment, the foregoing symptoms would continue from two to three weeks, when the epegastric uneasiness would subside, the appetite improve, the pulse return to its natural condition, the urine become more abundant, the intellect brighter, and the patient gradually resume his accustomed feelings of health, but often leaving the skin and eyes very yellow for several weeks. Such were the phenomena presented by the great majority of cases. But in some cases the symptoms of either general or local disturbance were exceedingly slight; the cases presenting very few *positive* symptoms except the deeply jaundiced hue of the skin. In a still smaller number of cases both the local and general symptoms were more severe. The epegastric tenderness and pain were more marked, the pulse quicker, the skin drier, and a deeper or more *bronzed* color, and a great part of all the food and drink taken would be rejected by vomiting in one or two hours after it was swallowed. In such, there was a feeling of great prostration, so much so as to keep the patients in bed for several days. Out of about sixty cases that occurred in my own practice, only one proved fatal. Concerning this last, I find the following notes recorded at the time.

November 28th, called to see Mrs. S., an American woman, aged thirty years, the mother of several children, and now about 4 months advanced in pregnancy. Says she has been sick three or four days with moderate diarrhœa, and ocasional vomiting. At present her skin and eyes are quite yellow; her tongue covered with a yellowish white fur; pulse 110 per minute, and soft; skin warmer than natural; urine scanty and high colored; intellect dull, and yet the patient sleepless and restless, with much thirst, burning in the stomach, no appetite, and the fecal discharges liquid and clay colored. The epigastric region was also slightly distended, tender to pressure and tympanitic. She was directed a powder composed of the sub. murias hydrarg. 2 grs. and sulph. morphine $\frac{1}{8}$ gr., every three hours, and a large mustard sinapism to to the epegastrium.

29th. The bowels had remained quiet since yesterday, but the paroxysms of vomiting had been more frequent. Directed a solution of sulph. morphine and soda, to be given in small doses and at short intervals, to quiet the stomach; and also fomentations to be applied to the abdomen.

30th. Vomiting less frequent; no evacuations from the bowels; pulse 130 per minute, full but soft; expression of countenance anxious with extreme restlessness, the patient to use her own language, feeling *as though she must fly:* abdomen moderately tympanitic; no secretion of urine; skin dry and very yellow. Directed a powder of calomel 10 grs., and Bi Carb. Soda 5 grs., to be given at once and followed in two hours by enemas of warm water and salt to move the bowels.

Dec. 1st. No improvement; no operation on the bowels; no urine; skin a deep bronzed yellow color, hotter then natural and dry; the ejections from the stomach mostly mucous and grass-green color; pulse 150 per minute and soft, mind dull, but great restlessness and thirst; abdomen distended, but bladder empty. Directed 10 grs. of calomel to be given in half an ounce of castor oil, to be followed by an enema of castor oil and oil of turpentine. Before the latter was administered, however, labor pains suddenly came on, and in a few minutes expelled a three months fœtus, which was followed by very moderate flowing, but all entirely without the consciousness of the patient. During the six hours following the labor the patient was more quiet; pulse less frequent but feeble; vomited but little; had two thin fecal discharges nearly destitute of any appearance of bile; discharges also a very small quantity of urine; but mind remained dull, approaching a comatose state. Directed powders of calomel, camphor and quinia in moderate doses.

Dec. 2d., 8 o'clock A. M. Patient more comatose; pulse 160 per minute, small and feeble; skin a shining bronze color, with an appearance of bloating all over; no further discharges either from the kidneys or bowels, but the frequent ejection of large quantities of a black or dark brown liquid, much like coffee grounds. Directed Chloride of Sodium in doses of one cruple every two hours, and 30 drops of oil of turpentine in the form of an emulsion between each dose of the salt.

In about three hours she passed a pint of reddish yellow urine, and soon after had two or three involuntary discharges from the bowels, looking much like that ejected from the stomach. From this time the vomiting ceased, but she died completely comatose during the following night.—No post-mortem examination could be obtained.—This case certainly presented some of the more importat features of genuine Yellow Fever. But from the manner of the attack, the early appearance of Jaundice, &c., I have no hesitation in regarding as simply a more severe form of the same epidemic that had just previously began to show itself throughout the community. The disease was restricted to no particular age or sex, though nearly three-fourths of all the cases that came under my observation occurred in persons in the early period of adult life, and who had been residents of this city *less* than one year. In regard to its Pathology, I think the early disturbance of the latter period of digestion; the sense of fulness with dull pain a little to the right and below the epegistrium, with more or less tenderness in the same region; the moderate febrile action, with clay colored fœces, and occasional nausea or vomiting; all indicate the existence of a low grade of inflammatory action located essentially in the trunk and ramifications of the biliary duets, and often extending to the mucous membrane of the Duodenum. This by obstructing the flow of bile into the intestine causes it to engorge the structure of the liver and return into the mass of the circulation, and thereby communicating a jaundiced hue to all the structures of the body.

How far the unusual warmth and extreme humidity of the atmosphere during the period when the foregoing disease made its appearance, acted as a predisposing or exciting cause, each member of the society must judge for himself.—That such a state of the atmosphere greatly lessens the excretion of the carbonic acid and aqueous vapor from the lungs and skin there can be no doubt. And as the liver is the next most important organ for eliminating carbonaceous matter, we can readily perceive that its secreting structure might be over excited, while at the same time the bile produced might be rendered morbid or more irritant than usual, inducing irritation and inflammation in the gall-ducts and duodenum; while the establishment of the latter would directly obstruct the further flow, and thereby rapidly induce the jaundice with all the subsequent phenomena. But I must hasten this report to a conclusion. During the same warm and wet month of December, two cases of well marked cholera were admitted into the Mercy Hospital, in a state of collapse, and a third in the earlier period of the disease. The two first were females. In both reaction was fully established. But one after progressing very favorably two days, was suddenly attacked with phlegmonous erysipelas of the neck and throat, and died in 48 hours. She was attacked with cholera in an emigrant car on her way from New Jersey to this city. The other woman passed into a secondary typhoid fever, from which she recovered in little more than two weeks. The third case was a child 18 months old, belonging to the woman that died of erysipelas. The child recovered. During the month of December also several cases of diarrhœa, relapsing agues, and typhoid pneumonias were met with. The latter, however, became decidedly more frequent on the first occurrence of steady cold weather during the first half of January.

From the foregoing review, drawn of course from my own personal circle of practice and observations, it will be seen that the year 1853 was characterized by the continuance of unusual immunity from all severe and fatal diseases in our city. In June, 1852, the population of the city was 38,733, and on the first of January, 1854, it was 60,600. The whole number of deaths during the year 1853 was reported to be 1,206.

If we suppose the average population of the city for the year 1853, to have been 55,000, the ratio of mortality was little less than 1 in 45½, which is a trifle less than that of Philadelphia, and very much less than that of Boston or New-York for the same year.

In conclusion, I would ask for the consideration of the society the following questions, viz:

1st. How far can we attribute the immunity from cholera and all other severe diseases during the warm season, to the unusual dryness of the atmosphere throughout the North-West during the months of June, July, and August?

2nd. The excessive heat of the middle of August was marked by great fatality in New-York and other eastern cities, while here there was scarcely a perceptible increase though the temperature wasnearly the same. Was this owing to the greater accompanying humidity along the Atlantic coast, as shown by the observations detailed in the first part of this paper?

